# Lung Metastasectomy from Colorectal Cancer, 10-year Experience in a South American Cancer Center

**DOI:** 10.3389/fsurg.2022.913678

**Published:** 2022-05-13

**Authors:** Carlos Carvajal, Helena Facundo, Paola Puerto, José Carreño, Rafael Beltrán

**Affiliations:** ^1^ Thoracic Surgery, National Cancer Institute, Bogota, Colombia; ^2^ Gastro-intestinal surgery, National Cancer Institute, Bogota, Colombia; ^3^ Oncologic surgery, National Cancer Institute, Bogota, Colombia; ^4^ Research Department, National Cancer Institute, Bogota, Colombia

**Keywords:** metastasectomy, colorectal neoplasms, neoplasm metastasis, multiple pulmonary nodules, solitary pulmonary nodule

## Abstract

**Purpose:**

This study aimed to describe the survival outcomes and factors associated with prognosis in patients undergoing pulmonary metastasectomy with colorectal cancer (CRC) in a cancer center in South America.

**Material and methods:**

A retrospective analysis of patients that underwent lung metastasectomy due to CRC at National Cancer Institute (INC), Colombia between 2007 and 2017 was performed and Kaplan-Meier survival analysis along with log-rank test and Cox regression multivariate analysis were completed.

**Results:**

Seventy-five patients with colorectal adenocarcinoma were included in the study. Of them, 57.3% were women with a median age of 62 years [interquartile ranges (IQR): 18.5]. For 45.3% the adenocarcinoma was located in the rectum and 29.3% had stage IV at diagnosis. 56% had a history of controlled extrapulmonary metastasis and 20% of the cases had a history of the metastasis of the liver. The median follow-up was 36.8 months (IQR: 27.4). Three-year and five-year overall survival (OS) was 57.5% [95% confidence interval (CI), 47.0–70.4] and 33.2% (95% CI, 23.4–47.2), respectively. Patients with bilateral, more than one pulmonary metastasis, abnormal postmetastasectomy carcinoembryonic antigen (CEA), history of liver metastasis, and disease-free interval (DFI) ≤12 months had worse OS. Three-year and five-year disease-free survival (DFS) was 30.1% (95% CI, 20.8–43.6) and 21.6% (95% CI, 13.0–35.9), respectively. Bilateral, more than one pulmonary metastasis, and patients with stage IV at diagnosis had the worst DFS. Multivariate analysis in the Cox regression model showed that abnormal postmetastasectomy CEA [Hazard Ratio (HR):1.97, 95% CI, 1.01–3.86, *p* = 0.045] and DFI ≤ 12 months (HR: 3.08, 95% CI, 1.26–7.53, *p* = 0.014) were independent factors for worst OS.

**Conclusions:**

The OS found falls within the wide range described in the world literature but interestingly it falls at the bottom end of this range. The factors associated with worst survival were identified as bilateral, more than one pulmonary metastasis, abnormal postmetastasectomy CEA, history of liver metastasis, and DFI ≤12 months.

**Contribution to the field:**

Pulmonary metastasectomy is the standard of care in patients with metastatic CRC. However, the literature supporting this conduct is based on retrospective studies and the only randomized controlled trial conducted to date was stopped due to poor recruitment. Limited information is available in South America about survival and factors associated with prognosis in patients with metastatic CRC. While this study is another series that adds to the many studies across the world that describe the use of pulmonary metastasectomy in CRC, it presents critical data as it is one of the few studies carried out in South America. As described in a wide range of world literature, OS found falls in patients that underwent lung metastasectomy due to CRC however; interestingly, in the South American population analyzed here it falls at the bottom end of this range. This may be explained by a large number of patients included with a history of extrapulmonary metastasis as well as may reflect inadequate patient access to reference cancer centers in Colombia. Factors associated with worst survival in our population were bilateral, more than one pulmonary metastasis, abnormal postmetastasectomy CEA, history of liver metastasis, and interval from diagnosis to development of pulmonary metastasis ≤12 months.

## Introduction

Based on the estimated new cancer cases for 2022, colorectal cancer (CRC) is the second highest cause of cancer deaths in the United States and the third most frequently occurring cancer in women and men (1). The lung is the second most common site of metastasis in patients with CRC. Although rectal cancer has a higher incidence of lung spread than colon cancer, nearly 20% of patients with CRC will develop lung metastasis (2).

General indications for pulmonary metastasectomy also apply for metastasectomy in CRC. These include adequate cardiopulmonary reserve that allows resection, controlled primary tumor, completely resectable lung metastases, no other effective treatment other than resection, and no extrapulmonary metastatic disease, or if present, controllable by surgery or other local therapy (3). Pulmonary metastasectomy has been generally accepted as a potentially curative option in the management of metastatic CRC. When metastasectomy is not feasible, the main treatment methods are chemotherapy and targeted therapy (4). The five-year survival rates of patients with metastatic CRC who receive only supportive care are less than 5%, while a systematic review and meta-analysis including 25 non-randomized studies published between 2000 and 2011, reported a five-year survival after complete resection between 27%–68% for pulmonary metastasectomy (5).

Limited information is available in South America about the role of pulmonary metastasectomy in metastatic CRC. Therefore, this study aimed to describe the survival outcomes and factors associated with prognosis in patients undergoing pulmonary metastasectomy with CRC in a cancer center in South America.

## Materials and Methods

We conducted a retrospective analysis of patients that underwent lung metastasectomy from colorectal cancer at the National Cancer Institute (INC), Colombia between December 1, 2007, and November 30, 2017. Exclusion criteria included patients with an uncontrolled extrapulmonary disease or who received other pulmonary local treatment modalities such as radiofrequency ablation or stereotactic radiotherapy. Medical records of all eligible patients were reviewed. This included evaluating demographic and histopathological data, characteristics of the primary tumor, staging, levels of carcinoembryonic antigen (CEA), characteristics of pulmonary metastases, surgical procedure, complications, and outcomes. Data were collected using the RedCap 7.1.2 platform.

Overall survival (OS) was calculated from the date of pulmonary metastasectomy until death or the last contact at the INC. Disease-free survival (DFS) was calculated from the date of the first pulmonary metastasectomy until documentation of disease recurrence by imaging or histopathologic confirmation. Disease-free interval (DFI) was calculated from the date of surgical management of the primary CRC until the date of the pulmonary metastasectomy. The normal serum CEA concentration range was 0–5 ng/mL. CEA prior to metastasectomy was performed up to six months before pulmonary metastasectomy and postmetastasectomy CEA was performed up to six months after. Numerical variables were presented in medians and interquartile ranges (IQR), while the categorical variables were presented in absolute values and percentages. Chi-square test was used to find associations between categorical variables and Wilcoxon signed-rank test for quantitative variables. Survival curves were performed using the Kaplan-Meier method and were compared by the log-rank test. A Cox regression model was performed for multivariate analysis that included statistically significant variables in the univariate analysis and clinical variables considered high risk in the literature. *p *≤ 0.05 was considered statistically significant. Statistical analysis was carried out using software R – Project v4.1.1. The study was approved by the institutional ethics and research committee (N° CEI-00855-20) and the data obtained were reviewed by an independent monitoring group.

## Results

Between 2007 and 2017, 75 patients underwent pulmonary metastasectomy from CRC, of which 43 (57.3%) were women and the median age was 62 years (IQR: 18.5). Regarding the characteristics of the primary tumor, 80.0% of the cases were usual adenocarcinoma, followed by mucinous adenocarcinoma in 17.3% and Signet-ring cell adenocarcinoma in 2.7%; half of the patients included were stage III (53.3%) and 29.3% had stage IV at diagnosis (Table 1). A moderate degree of differentiation was described in 62.7% of the patients and the most frequent location was the rectum (45.3%); Thirty (40%) patients had more than one pulmonary metastasis, half of the metastases were unilateral (52.0%) (Table 2) and 56.0% of the patients had a history of extrapulmonary metastasis, the liver being the most frequent location (20.0%), followed by peritoneum (13.3%) and retroperitoneum (6.7%) (Table 1). PET-CT was not a routine procedure before pulmonary metastasectomy in CRC, in our service.

**Table 1:** Demographic and clinical characteristics.

Characteristic			Total (*n* = 75)
Age (years)	Median [IQR^a^]		62.0 [18.5]
Gender, *n* (%)	Male		32 (42.7)
	Female		43 (57.3)
Stage, *n* (%)	I		2 (2.70)
	II		11 (14.7)
	III		40 (53.3)
	IV		22 (29.3)
Primary tumor histology, *n* (%)	Mucinous adenocarcinoma		13 (17.3)
	Usual adenocarcinoma		60 (80.0)
	Signet-ring cell		2 (2.70)
History of extrapulmonary metastasis, *n* (%)			42 (56.0)
Liver	15 (20.0)		
Peritoneum	10 (13.3)		
Retroperitoneum	8 (10.7)		
Ovary	4 (5.4)		
Bone	3 (4.0)		
Inguinal Lymph node	1 (1.3)		
Central nervous system	1 (1.3)		
Number of pulmonary procedures, *n* (%)	1		42 (56.0)
	2		26 (34.7)
	3–4		7 (9.30)
Mutation Analysis: (*n* = 63)	MMR^b^	5(7.9)^c^	
	B-RAF	1(1.6)	
	K-RAS	35(55.5)^d^	
	N-RAS	4(6.3)	
Recurrence, *n* (%)	Yes		53 (70.7)
	No		22 (29.3)
Death, *n* (%)	Yes		50 (66.7)
	No		25 (33.3)

^*a*^ *IQR: Interquartilic range.*

^*b*^ *Includes: MLH1, MSH2, MSH6, PMS2.*

^*c*^ *One patient had MMR and N-RAS mutation.*

^*d*^ *Two patients had K-RAS and N-RAS mutation.*

**Table 2:** Association between variables and death.

Variables		Total (*n* = 75)	Death (*n* = 50)	Alive (*n* = 25)	*p*-value^a^
Degree of differentiation, *n* (%)	Well	20 (26.7)	14 (28.0)	6 (24.0)	0.233
	Moderate	47 (62.7)	30 (60.0)	17 (68.0)	
	poor	5 (6.60)	5 (10.0)	0 (0.00)	
	No data	3 (4.00)	1 (2.00)	2 (8.00)	
Localization, *n* (%)	Ascending colon	16 (21.4)	9 (18.0)	7 (28.0)	0.595
	Descending colon	25 (33.3)	17 (34.0)	8 (32.0)	
	Rectum	34 (45.3)	24 (48.0)	10 (40.0)	
CEA^b^ prior to metastasectomy	Median [IQR^c^]	3.59 [5.60]	4.40 [6.60]	2.63 [4.34]	0.154^d^
Postmetastasectomy CEA	Median [IQR]	3.72 [8.72]	7.98 [15.5]	1.96 [1.90]	<0.01^d^
Pulmonary metastasis, *n* (%)	Unilateral	39 (52.0)	22 (44.0)	17 (68.0)	0.048
	Bilateral	36 (48.0)	28 (56.0)	8 (32.0)	
Size of metastases,^e^ *n* (%)	Median [IQR]	10.7 [9.50]	10.4 [8.75]	13.0 [8.00]	0.513
Number of pulmonary metastases, *n* (%)	1	44 (58.7)	26 (52.0)	18 (72.0)	<0.01
	2–3	23 (30.7)	18 (36.0)	5 (20.0)	
	>3	7 (9.30)	5 (10.0)	2 (8.00)	
	No data	1 (1.30)	1 (2.00)	0 (0.00)	
Number of pulmonary procedures, *n* (%)	1	42 (56.0)	27 (54.0)	15 (60.0)	0.666
	2	26 (34.7)	19 (38.0)	7 (28.0)	
	3–4	7 (9.30)	4 (8.00)	3 (12.0)	
Disease free survival	≤12 months	17 (22.7)	15 (30.0)	2 (8.00)	0.032
	>12 months	58 (77.3)	35 (70.0)	23 (92.0)	
Chemotherapy, *n* (%)	Yes	71 (94.7)	47 (94.0)	24 (96.0)	1.00
	No	4 (5.30)	3 (6.00)	1 (4.00)	

^*a*^ *Chi square test.*

^*b*^ *CEA: carcinoembryonic antigen.*

^*c*^ *IQR: interquartilic range.*

^*d*^ *Wilcoxon signed-rank test*

^*e*^ *Largest diameter of the biggest metastasis.*

Mutation analysis was performed in 63 (84.0%) patients. Of these, the most frequent mutation was Kirsten rat sarcoma virus (K-RAS) in 55.5%, including two patients with K-RAS and Neuroblastoma rat sarcoma virus (N-RAS) mutations (Table 1).

In our series, 42 (56.0%) patients underwent one surgical lung procedure, 26 (34.7%) two procedures, and seven (9.3%) three or four procedures (Table 1). A total of 117 surgical procedures were performed on these 75 patients. Wedge resection was the main pulmonary resection performed in all surgical procedures, corresponding to 70.1%, followed by lobectomy in 13.7%. Video-assisted thoracoscopic surgery (VATS) was the approach in 91 procedures (77.8%), of which 65.9% were uniportal VATS and 34.1% were multiportal VATS; conversion rate was 5.5%. A mediastinal lymph node sample was performed in 17.3% of patients, and only two patients (2.7%) were positive for metastasis. Meanwhile, a hilar lymph node sample was performed in 8.0% of patients and only one patient presented tumor involvement. A pulmonary artery injury was the only intraoperative complication described during a lobectomy, there was no intraoperative mortality and the postoperative complication rate was 5.1%. Regarding the systemic treatment of the primary CRC, 94.7% of patients received chemotherapy: neoadjuvant in 5.30%, adjuvant in 58.7%, and neoadjuvant/adjuvant in 30.7%. The median DFI was 25.6 months (IQR: 25.1).

**Figure 1:**
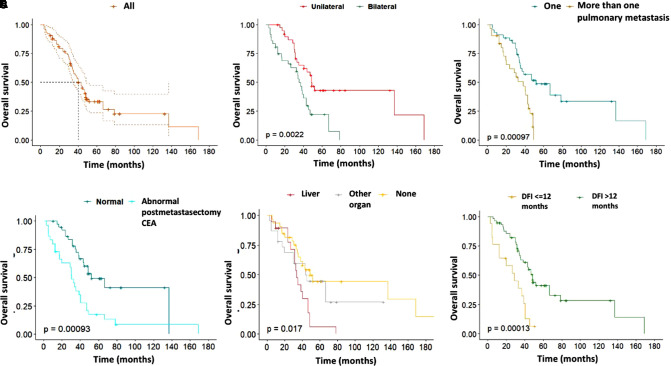
(**A**) Overall survival (OS). (**B**) OS of patients with unilateral and bilateral metastases. (**C**) OS according to the number of pulmonary metastases (**D**) OS of patients with normal and abnormal postmetastasectomy carcinoembryonic antigen (**E**) OS of patients with a history of extrapulmonary metastasis (**F**) OS according to the disease-free interval ≤12 and >12 months. Kaplan-Meier curves with the log-rank test.

The median follow-up was 36.8 months (IQR: 27.4) and 50 (66.7%) patients died during this period. Three-year and five-year OS was 57.5% (95% CI, 47.0–70.4) and 33.2% (95% CI, 23.4–47.2), respectively. The median OS was 40.4 months (95% CI, 35.1–48.8) (Figure 1A).

The Kaplan-Meier curves showed that patients with bilateral (*p* = 0.0022), more than one pulmonary metastasis (*p* = 0.0009), abnormal postmetastasectomy CEA (*p* = 0.0009), history of liver metastasis (*p* = 0.0170) and DFI ≤12 months (*p* = 0001) had a worse OS (Figures 1B–F) and these differences were statistically significant (*p* < 0.01) (Table 3).

**Table 3:** Associations between overall survival, disease-free survival and clinical variables.

	Overall Survival % [CI_95%_]		*p*-value^a^
	36 months	60 months	
Metastasis			
Unilateral	64.8 [51.1–82.2]	43.1 [29.2–63.6]	0.0022
Bilateral	49.6 [35.1–70.1]	22.2 [11.4–43.7]	
Number of pulmonary metastases			
One	61.9 [48.8–78.6]	48.7 [35.4–67.1]	0.0009
More than one	50.6 [35.1–73.1]	–	
Postmetastasectomy CEA^b^			
Normal	72.4 [59.2–88.6]	49.3 [34.6–70.5]	0.0009
Abnormal	38.4 [24.2–60.9]	17.5 [7.89–38.7]	
Extrapulmonary metastasis			
Liver	41.8 [23.8–73.3]	5.96 [0.89–39.8]	0.0170
Other	59.8 [42.6–84.1]	44.4 [28.0–71.9]	
None	64.9 [50.1–84.1]	44.9 [28.7–68.6]	
Disease free interval			
≤12 months	38.8 [21.0–71.9]	–	<0.01
>12 months	62.9 [51.2–77.3]	41.4 [29.6–57.9]	
	Disease Free Survival % [CI_95%_]		*p*-value^a^
	36 months	60 months	
Metastasis			
Unilateral	41.1 [27.5–61.7]	34.7 [21.9–55.0]	0.0026
Bilateral	18.5 [8.80–38.9]	13.8 [5.45–35.3]	
Number of pulmonary metastases			
One	58.0 [40.3–83.3]	47.4 [30.0–74.9]	0.0027
More than one	16.2 [8.15–32.4]	13.5 [6.23–29.5]	
Stage			
I,II and III	39.0 [27.4–55.6]	27.5 [16.6–45.6]	0.0048
IV^c^	5.91 [0.89–39.3]	–	

^*a*^ *Log-Rank test.*

^*b*^ *CEA: carcinoembryonic antigen.*

^*c*^ *Stage IV at diagnosis.*

Multivariate analysis showed that DFI ≤12 months (HR: 3.08, 95% CI, 1.26–7.53, *p* = 0.014) and abnormal postmetastasectomy CEA (HR:1.97, 95% CI, 1.01–3.86, *p* = 0.045) were independent factors for worst overall survival (Table 4).

**Table 4:** Univariate and multivariate analysis of overall survival.

Variables	Univariate		Multivariate	
	HR^a^ [IC_95%_]	*p*-value	HR^a^ [IC_95%_]	*p*-value
DFI^b^ (months)				
** **≤12	4.40 [2.16–8.99]	**<0**.**01**	3.08 [1.26–7.53]	**0**.**014**
** **>12	Ref.		Ref.	
Postmetastasectomy CEA^c^				
Normal	Ref.		Ref.	
Abnormal	2.98 [1.60–5.53]	**<0**.**01**	1.97 [1.01–3.86]	**0**.**045**
Extrapulmonary metastasis				
None	Ref.	** **	Ref.	
Liver	2.38 [1.14–4.96]	**0**.**020**	1.39 [0.56–3.48]	0.470
Other	1.28 [0.58–2.79]	0.532	0.99 [0.39–2.56]	0.988
K-RAS				
No mutation	Ref.		Ref.	
Mutation	1.17 [0.63–2.16]	0.623	0.93 [0.46–1.86]	0.835
Stage				
I-II-III	Ref.		Ref.	
IV	1.56 [0.84–2.91]	0.156	1.46 [0.64–3.34]	0.370
Metastasis				
Unilateral	Ref.	** **	Ref.	
Bilateral	2.28 [1.23–4.25]	**<0**.**01**	1.42 [0.60–3.33]	0.421
Number of pulmonary metastases				
One	Ref.	** **	Ref.	
More than one	2.30 [1.08–4.93]	**0**.**032**	1.23 [0.43–3.47]	0.698

^*a*^ *HR: Hazard Ratio.*

^*b*^ *DFI: Disease free interval.*

^*c*^ *CEA: carcinoembryonic antigen.*

**Figure 2:**
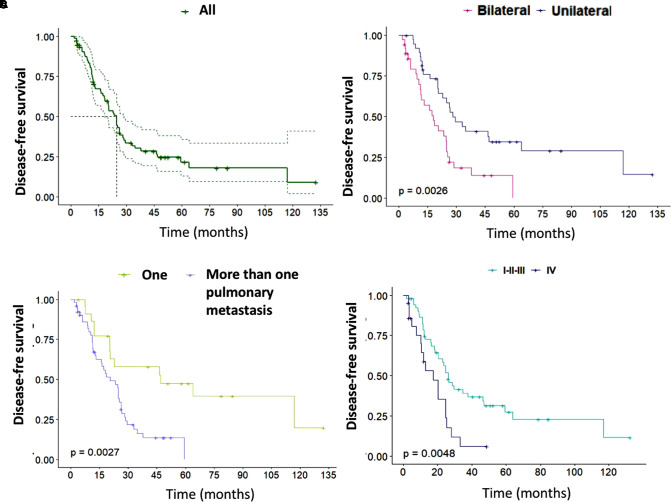
(**A**) Disease-free survival (DFS). (**B**) DFS of patients with unilateral and bilateral metastases. (**C**) DFS according to the number of pulmonary metastases. (**D**) DFS according to the stage at diagnosis. Kaplan-Meier curves with the log-rank test.

On the other hand, 53 (70.7%) patients presented disease recurrence during the follow-up period. Three-year and five-year DFS was 30.1% (95% CI, 20.8–43.6) and 21.6% (95% CI, 13.0–35.9), respectively. The median DFS was 24.6 months (95% CI, 18.5–28.6) (Figure 2A). Bilateral, more than one pulmonary metastasis, and patients with stage IV at diagnosis had the worst DFS (Figures 2B–D) (Table 3). There was no association between DFI ≤12 months and recurrence (*p* = 0.559).

## Discussion

Several retrospective studies support the use of pulmonary metastasectomy as a standard of care in metastatic CRC (6) but some do not show clear survival benefits at present (7). Our study found a five-year OS of 33.2%, which is within the range of OS in such cases described in the literature (27%–68%) (5). Nevertheless, it is far from the 49.8% found by Gössling in Brazil (8) and even further from the 68.1% described by Okumura in Japan (9). Our OS was similar to that reported in the PulMiCC trial (NCT01106261), the only randomized controlled trial to date comparing pulmonary metastasectomy with systemic therapy, which reported a 38% five-year survival for metastasectomy and was stopped due to poor recruitment (10). We believe, that the factors contributing to worse OS in our study when compared to other series were, a high number of patients with bilateral (48%), more than one metastasis (40%), and history of extrapulmonary metastasis (56%) in the population as well as the likelihood of inadequate access to reference cancer centers in Colombia.

In previous reports, five-year DFS in patients with CRC that underwent pulmonary metastasectomy ranged from 26.7% to 31% (11, 12). In our series, the five-year DFS was slightly below this range (21.6%) probably due to the factors described above. Nevertheless, it should be noted that the median DFS was 24.6 which was higher than the median DFS of 14 months (95% CI, 10.4–17.5) described by Gössling et al. (8), in their study that included 58 patients who underwent pulmonary metastasectomy from CRC in Brazil.

Additionally, several prognostic factors associated with poor survival in patients with metastatic CRC have been described. These include short DFI, multiple pulmonary metastases, involvement of hilar or mediastinal lymph nodes, elevated CEA before metastasectomy, tumor size, patients older than 70 years, extrapulmonary metastatic lesions treated before pulmonary metastasectomy, synchronous presentation, advanced primary tumor stage and rectum localization (3, 5, 6, 13).

As described in a 2013 metanalysis, short DFI is one of the most important factors of worst prognosis in pulmonary metastasectomy in CRC, as it was associated with an increased risk of death (HR: 1.59, 95% CI, 1.27–1.98) (5). In our series, we found an association between DFI ≤12 months and worst OS (HR: 3.08, 95% CI, 1.26–7.53, *p* = 0.014) in the multivariate analysis.

Fukada et al. (14) described that CEA before metastasectomy, in the univariate analysis, was one of the seven significant factors related to survival. In our series, we did not find an association between survival and CEA before metastasectomy but we did find an association in the univariate and multivariate analysis between worst OS and abnormal postmetastasectomy CEA (HR:1.97, 95% CI, 1.01–3.86, *p* = 0.045). Fukada et al. did not describe postmetastasectomy CEA or its association with survival.

Postmetastasectomy CEA is not a commonly mentioned finding in the literature, probably because few studies are focused on repeated lung resections for metastases and because postmetastasectomy CEA may correspond to CEA before the second procedure in those series where more than one surgery is performed. In our study, 44% of the patients underwent more than one procedure for pulmonary metastasectomy and we did not find an association between the number of procedures and death (*p* = 0.666). Ambrogi et al. (15) described that the lack of guidelines for redo pulmonary metastasectomy and the insignificant increment of perioperative mortality or morbidity, make redo metastasectomy a feasible procedure, especially in patients with metastatic CRC who represent the most common pathology in which redo metastasectomy is performed.

In our series, 20% of the patients had a history of controlled hepatic metastasis before pulmonary metastasectomy. The five-year OS of these patients was 6% and in the univariate analysis, these patients had worst overall survival compared to those patients without extrapulmonary metastasis and even in patients with other controlled extrapulmonary metastasis. Although five-year OS of patients with a history of hepatic metastasectomy before pulmonary metastasectomy ranges from 11 to 65% in the previous series (16, 17) and therefore is strongly recommended, our results were not so encouraging and must be taken into account when deciding to perform pulmonary metastasectomy.

Furthermore, Cho et al. (18) found that patients with multiple pulmonary metastases in CRC were associated with the worst prognosis (HR = 2.121, 95% CI, 1.081–4.159, *p* = 0.029); similar results in the univariate analysis were found in our series where HR = 2.30 (95% CI, 1.08–4.93, *p* = 0.032) was obtained.

Moreover, Socola et al. (19) described that bilateral metastases were found only in 7% of the patients that underwent resection in their retrospective cohort study that included 28 patients with pulmonary metastases managed with surgery and 46 controls managed without surgery. They also described that there was no association between bilateral pulmonary metastasectomy and survival (*p* = 0.717). Likewise, in our series, 48% of our patients had bilateral metastases and in the univariate analysis, we did find that patients with bilateral metastases had the worst overall survival (HR = 2.28, 95% CI, 1.23–4.25, *p* < 0.01).

Mediastinal lymph node metastasis was the main factor of the worst prognosis for survival after pulmonary metastasectomy in CRC found by Hamaji et al. (20) (*p* = 0.047 in univariate and *p* = 0.0028 in multivariate analysis) and they recommended the use of systematic mediastinal lymph node dissection for prognostic purposes. However, in our series, we did not find an association between positive hilar or mediastinal lymph node and worst prognosis. This may be explained by the fact that these are not routine procedures in our hospital practice, mediastinal and hilar lymph node sampling was performed in 17.3% and 8.0% of patients, respectively.

Gössling et al. (8) reported that variables that showed significance with long-term relapse-free survival and OS were longer DFI, large lesion size, number of lesions, metachronous presentation, and CK20 expression in metastases. But it must be noted that laterality and adjuvant or perioperative chemotherapy did not have a statistical impact in their sample. In our series, the variables associated with worst DFS were bilateral metastases, more than one pulmonary metastasis, and patients with stage IV at diagnosis had worst DFS.

The limitations of this study include its retrospective nature and single-center analysis. Medical records had incomplete information on variables such as the degree of differentiation and the number of metastases. Furthermore, complete information about the management of extrapulmonary metastases was not available and mutation analysis was not performed on the total population. Although this study is one more series of many in the world that describes the use of pulmonary metastasectomy in CRC, it presents some interesting insights, as it is one of the few studies that were carried out in South America.

## Conclusions

The overall survival found in this series falls within the wide range described in the world literature however; remarkably it falls at the bottom end of this range. Aspects that we believe contributed to our worse OS when compared to other series are a high number of patients included bilateral, more than one metastasis, and history of extrapulmonary metastasis. These findings may also reflect inadequate access to reference cancer centers in Colombia, a country that belongs to lower- and median-income countries.

Factors associated with worst survival were bilateral, more than one pulmonary metastasis, abnormal postmetastasectomy CEA, history of liver metastasis, and DFI ≤12 months.

Postmetastasectomy CEA is not a commonly mentioned finding in the literature, probably because few studies are focused on repeated lung resections for metastases and because postmetastasectomy CEA may correspond to CEA before the second procedure in those cases where more than one surgery is performed. These factors associated with the worst prognosis must be taken into account when deciding on the management of patients with metastatic CRC.

## Data Availability

The raw data supporting the conclusions of this article will be made available by the authors, without undue reservation.
